# Association Between C-Peptide Level and Subclinical Myocardial Injury

**DOI:** 10.3389/fendo.2021.680501

**Published:** 2021-08-12

**Authors:** Ziwei Chen, Jing He, Qiang Ma, Mingbing Xiao

**Affiliations:** ^1^Department of Cardiology, Affiliated Hospital of Nantong University, Nantong, China; ^2^Department of Oncology, Affiliated Hospital of Nantong University, Nantong, China; ^3^School of Public Health, Nantong University, Nantong, China; ^4^Research Center of Clinical Research Center of Clinical Medicine, Affiliated Hospital of Nantong University, Nantong, China

**Keywords:** C-peptide, subclinical cardiac injury, NHANES III, association, cross sectional study

## Abstract

**Background:**

Previous studies have confirmed an association between C-peptide levels with the risk of cardiometabolic diseases. However, whether circulating C-peptide was related to subclinical myocardial injury (SC-MI) remains unknown.

**Methods:**

A total of 3,752 participants without a history of cardiovascular diseases were included in our study from National Health and Nutrition Examination Survey III (NHANES III). Multivariable linear regression was performed to explore the correlation between C-peptide and cardiac injury score (CIIS). Multivariate logistic regression was used to examine the association between C-peptide quartile and SC-MI.

**Results:**

Circulating C-peptide was significantly associated with CIIS (β:0.09, 95% confidence interval [CI]: 0.00–0.17; *p* = 0.041). Compared with the lowest quartile, the highest quartile of circulating C-peptide increased a 1.48-fold risk of SC-MI (Odds ratio = 1.66, 95% CI: 1.18–1.87; *p* = 0.001).

**Conclusions:**

The level of C-peptide was independently associated with CIIS and SC-MI, which could serve as a new risk factor of SC-MI.

## Background

Subclinical myocardial injury (SC-MI) is an early cardiac injury without clinically evident coronary heart disease or heart failure (1, 2). SC-MI is defined by an electrocardiographic-based scoring system, namely, cardiac infarction/injury score (CIIS) >10 (3). SC-MI was reported to be associated with the progression of coronary heart disease (1) and cardiovascular and all-cause mortality (4). Previous studies have reported that physical activity (5), obesity (6), diastolic blood pressure (7), Vitamin D (8) and TyG index (9) were associated with SC-MI.

C-peptide is a small peptide with 31 amino acids and is released upon insulin secretion to ensure the correct folding of proinsulin (10, 11). It is known that C-peptide has been widely used as a biomarker of diabetes diagnosis in clinical practice. Observational studies have found the association between C-peptide and cardiovascular diseases (12, 13). High levels of C-peptide could increase the risks of atherosclerosis and myocardial infarction (14, 15). C-peptide was reported to increase the level of triglyceride and to decrease HDL-C (16). However, C-peptide also inhibited oxidative stress and endothelial apoptosis (17), showing a cardioprotective role. Therefore, it remains unknown that circulating C-peptide level was associated with SC-MI.

In our study, we examined the association between levels of serum C-peptide and SC-MI based on a cross-sectional study.

## Methods

### Study Population

**Figure 1:**
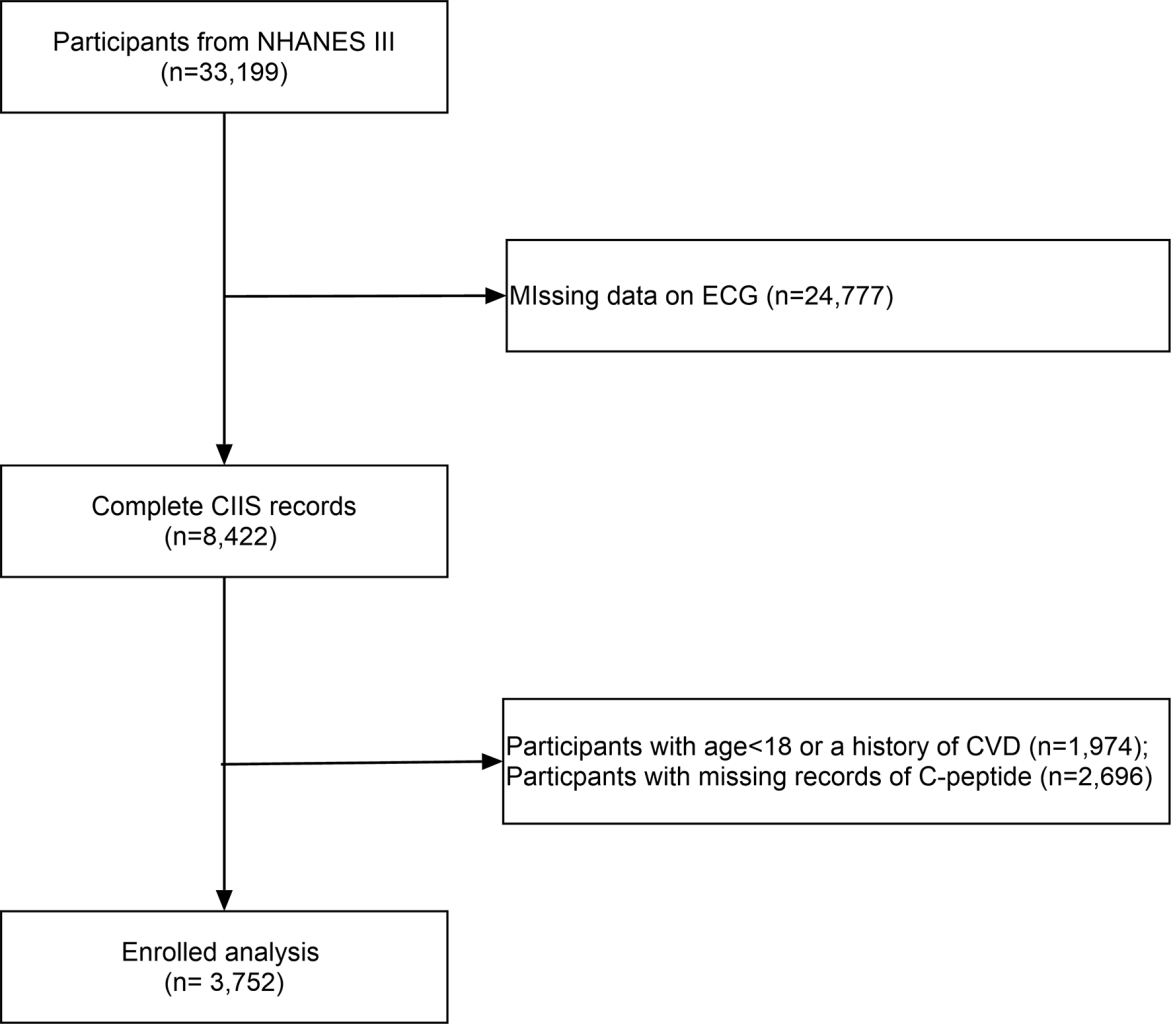
The flow chart of the selection process.

All participants were included from the US National Health and Nutrition Examination Survey (NHANES III). The NHANES is nationwide multistage survey designed to assess the health and nutritional status of adults and children in the United States (https://www.cdc.gov/nchs/nhanes/index.htm) by the Centers for Disease Control and Prevention (CDC). After excluding individuals with missing circulating C-peptide data, we included 3,752 participants without a history of cardiovascular diseases (**Figure 1**). The survey protocol was approved by the Institutional Review Board of the CDC.

### Covariate Assessments

The list of covariates includes baseline demographics, the risk factor of cardiovascular diseases, or factors influencing C-peptide levels. Sociodemographic variables, including age, gender, and race, were collected by using standardized questionnaires. The systolic blood pressure, diastolic blood pressure, pulse rate, height, and weight of each participant were obtained from the physical examinations. White blood cell (WBC), red blood cell (RBC), and hemoglobin were obtained by whole blood cell count test. Triglyceride (TG), total cholesterol (TC), low-density lipoprotein cholesterol (LDL-C), high-density lipoprotein cholesterol (HDL-C), C-reactive protein (CRP), creatinine, alanine aminotransferase (ALT), aspartate transaminase (AST), glucose, glycated hemoglobin, and insulin were measured by standard biochemistry assays. Body mass index (BMI, kg/m^2^) was calculated as weight divided by height squared. Race was classified as non-Hispanic white, non-Hispanic black, Mexican American, and other. Smokers were defined as those who self-reported smoking more than 100 cigarettes during their lifetime, and alcohol users were those who had at least 12 drinks in the last 12 months (18). Physical activity was defined as taking vigorous or moderate activities. Multiple imputation was performed for covariates with missing values.

### Circulating C-Peptide Measurement

Serum C-peptide was measured using a radioimmunoassay (RIA), where the 125I-labeled C-peptide completes with C-peptide in the specimen for antibody site. Bound and free C-peptide is separated by adding a second PEG-accelerated double antibody. The antibody-bound fraction is precipitated and counted. The radioactivity is inversely proportional to the quantity of C-peptide in the specimen. Circulating C-peptide was treated as a continuous and a quartile variable to examine its association with CIIS and SC-MI, respectively. The quartile of C-peptide levels was categorized into four groups: Q1 (<0.452 nmol/L), Q2 (0452–0.737 nmol/L), Q3 (0.737–1.082 nmol/L), and Q4 (>1.082 nmol/L).

### Outcome Definition

Resting 12-lead electrocardiograms were recorded by experienced technicians with a Marquette MAC 12 system. Analysis of electrocardiograms was achieved through a computerized automated process and visual inspection by a trained technician located in a centralized core laboratory. Briefly, the SC-MI defined from the CIIS rests on a weighted scoring system taking several objective electrocardiographic waveform components related to myocardial injury and ischemia, both discrete and continuous, and generating a risk-stratified scoring system (6, 7). A combination of 11 discrete and 4 continuous variables are counted to define the final score to evaluate the disease severity levels: CIIS>20 for probable injury; CIIS>15 for possible injury; CIIS>10 for borderline abnormality. SC-MI was defined as a total of CIIS>10 (3).

### Statistical Analysis

Sample weights and stratification were incorporated in all analyses because of the complex sampling design of the NHANES data. Categorical variables were expressed as frequencies and percentages. Continuous variables were reported as mean ± standard deviation or median (interquartile range) if skewed distribution. The difference between groups were compared using one-way ANOVA for continuous variables or Kruskal–Wallis test if not normalized distributed and chi-square test for categorical variables. The multivariable linear regression was used to explore the association between C-peptide and CIIS (log2-transformed for normality) while the multivariable logistic regression was used to explore the association between C-peptide and SC-MI. To rule out the cofounding factors, we adjusted for age and gender in Model 1. In model 2, we adjusted for age, gender, race, smoking, drinking, smoking, physical activity, and BMI. In model 3, we adjusted for age, gender, race, smoking, drinking, smoking, BMI, TC, TG, CRP, creatinine, AST, ALT, glucose, glycated hemoglobin, and insulin. The variables that were adjusted for were based on the *p* value <0.05 in univariate analysis or the risk factors of cardiometabolic diseases. The restricted cubic spline models with knots at 10th, 50^th^, and 90th percentage were used for the dose–response analysis. Data were analyzed using IBM SPSS 25.0 and R software 3.6. A two-tailed *p*-value <0.05 was considered as statistically significant.

## Results

### Baseline Characteristics

A total of 3,752 participants were enrolled in our study with an average age of 60.2 ± 13.1, of which 1,778 (47.3%) were males. **Table 1** showed the baseline characteristics grouped by quartiles of circulating C-peptide levels. Participants with high C-peptide levels tend to be old, and male, as well as having higher levels of BMI, TC, TG, LDL-C, CRP, glucose, and glycated hemoglobin.

**Table 1:** Baseline characteristics of study participants across serum C-peptide categories.

	Q1 (≤0.452)	Q2 (0.452–0.737)	Q3 (0.737–1.082)	Q4 (≥1.082)	*p*-value
***n***	941	938	936	937	
Age (years)	58.4 ± 13.3	59.6 ± 12.9	61.3 ± 13.4	61.4 ± 12.8	<0.001
Male (%)	377 (40.1)	447 (47.7)	472 (50.4)	480 (51.2)	<0.001
Race (%)					<0.001
Non-Hispanic white	521 (55.4)	527 (56.2)	484 (51.7)	476 (50.8)	
Non-Hispanic black	249 (26.5)	198 (21.1)	200 (21.4)	172 (18.4)	
Mexican-American	142 (15.1)	179 (19.1)	220 (23.5)	252 (26.9)	
Other	29 (3.1)	34 (3.6)	32 (3.4)	37 (3.9)	
Smoking (%)	312 (33.2)	303 (32.3)	265 (28.3)	202 (21.6)	<0.001
Drinking (%)	511 (54.3)	523 (55.8)	549 (58.7)	523 (55.8)	0.467
Physical activity (%)	580 (61.7)	513 (54.7)	478 (51.1)	461 (49.2)	<0.001
BMI (kg/m^2^)	24.0 ± 4.2	26.5 ± 4.2	28.5 ± 5.2	31.2 ± 6.1	<0.001
sBP (mmHg)	127.6 ± 19.8	130.9 ± 18.6	134.9 ± 19.1	136.2 ± 18.3	<0.001
dBP (mmHg)	58.3 ± 6.8	58.1 ± 7.1	58.1 ± 7.2	57.9 ± 7.4	0.680
Pulse (bpm)	73.3 ± 11.4	74.7 ± 11.9	76.0 ± 12.3	79.0 ± 13.1	<0.001
WBC, 10^9^/L	6.6 ± 2.0	6.9 ± 2.2	7.3 ± 3.1	7.8 ± 2.6	<0.001
RBC, 10^12^/L	4.5 ± 0.4	4.6 ± 0.4	4.7 ± 0.4	4.8 ± 0.5	<0.001
Hemoglobin (g/dl)	13.5 ± 1.4	14.0 ± 1.4	14.1 ± 1.4	14.4 ± 1.5	<0.001
Total cholesterol (mg/dl)	206 (53)	216 (51)	216 (52)	214 (52)	<0.001
Triglycerides (mg/dl)	86 (49)	113 (61)	139 (86)	166 (91)	<0.001
LDL-C (mg/dl)	127 (49)	140 (44)	138 (47)	136 (47)	<0.001
HDL-C (mg/dl)	57 (23)	51 (20)	46 (16)	42 (14)	<0.001
C-reactive protein (mg/dl)	0.21 (0.1)	0.21 (0.2)	0.21 (0.3)	0.33 (0.5)	<0.001
Creatinine (mg/dl)	1.05 ± 0.18	1.08 ± 0.22	1.11 ± 0.27	1.18 ± 0.55	<0.001
AST (U/L)	21.7 ± 13.8	21.2 ± 11.6	22.3 ± 16.9	24.6 ± 18.7	<0.001
ALT (U/L)	13.6 ± 9.9	15.1 ± 9.5	17.6 ± 14.2	21.7 ± 17.8	<0.001
Glucose (mg/dl)	90 (13)	96 (14)	101 (17)	108 (24)	<0.001
Glycated hemoglobin (%)	5.5 ± 1.0	5.7 ± 1.1	5.9 ± 1.3	6.2 ± 1.5	<0.001
Insulin (µU/ml)	5.6 (2.5)	7.9 (2.9)	11 (4.4)	17.8 (9.3)	<0.001
CIIS	6.2 (8.7)	6.6 (7.8)	7.2 (9.6)	8.5 (11.2)	<0.001
SC-MI	297 (31.6)	646 (31.1)	361 (38.6)	528 (43.6)	<0.001

### Association Between C-Peptide Levels and CIIS

The CIIS was higher with the increased quartile of C-peptide. Multivariable linear regression analysis was used to explore the association between C-peptide and log2-transformed CIIS (**Table 2**). After adjusting for age and sex, circulating C-peptide was positively related to CIIS (*β* = 0.13, 95% CI: 0.06–0.19; *p* < 0.001). The linear relationship still existed after adjusting for lifestyles in Model 2 (*β* = 0.09, 95% CI: 0.00–0.17; *p* = 0.039) and laboratory examinations in Model 3 (*β* = 0.09, 95% CI: 0.00-0.17; *p* = 0.041). Compared with the lowest quartile, the highest quartile of C-peptide was positively associated with CIIS across three models.

**Table 2:** Multivariable linear regression between C-peptide and log2-CIIS.

	Model 1		Model 2		Model 3	
	*β* (95% CI)	*p*	*β* (95% CI)	*p*	*β* (95% CI)	*p*
Q1	Ref		Ref		Ref	
Q2	0.01 [−0.10, 0.11]	0.892	−0.01 [−0.11, 0.10]	0.863	−0.01 [−0.11, 0.10]	0.856
Q3	0.13 [0.02, 0.23]	0.015	0.10 [−0.01, 0.21]	0.074	0.10 [−0.01, 0.21]	0.083
Q4	0.21 [0.10, 0.31]	<0.001	0.16 [0.04, 0.28]	0.011	0.16 [0.03, 0.28]	0.015
Per one-unit	0.13 [0.06, 0.19]	<0.001	0.09 [0.00, 0.17]	0.039	0.09 [0.00, 0.17]	0.041

OR, odds ratio; CI, confidence interval; BMI, body mass index; TC, total cholesterol; TG, triglyceride, CRP, c-reactive protein.

Model 1 was adjusted for age and gender.

Model 2 was adjusted for age, gender, race, smoking, drinking, physical activity, BMI, TC, TG, CRP, and creatinine.

Model 3 was adjusted for age, gender, race, smoking, drinking, physical activity, BMI, TC, TG, CRP, creatinine, glucose, glycated hemoglobin, and insulin.

### Association Between C-Peptide Levels and SC-MI

The prevalence of SC-MI was 31.6%, 31.3%, 38.6%, and 43.6% across quartiles, respectively. **Table 3** summarizes the results of multivariable logistic regression between C-peptide quartiles and SC-MI. Compared to the lowest quartile, the highest quartile was significantly associated with SC-MI in model 1 adjusted for sociodemographics (OR = 1.59, 95% CI:1.31–1.92; *p* < 0.001), and this association remained statistically significant in Model 2 (OR = 1.48, 95% CI:1.18–1.86; *p* = 0.001) and Model 3 (OR = 1.48, 95% CI:1.18–1.87; *p* = 0.001). In addition, one-unit increase of C-peptide was associated with 1.27-fold higher risk of SC-MI (OR = 1.27, 95% CI:1.08–1.50; *p* = 0.004).

**Table 3:** Multivariable logistic regression between C-peptide categories and subclinical myocardial injury.

	Model 1		Model 2		Model 3	
	OR (95% CI)	*p*	OR (95% CI)	*p*	OR (95% CI)	*p*
Q1	Ref		Ref		Ref	
Q2	0.95 [0.78, 1.16]	0.619	0.92 [0.75, 1.13]	0.426	0.92 [0.75, 1.12	0.414
Q3	1.29 [1.06, 1.56]	0.010	1.23 [1.00, 1.51]	0.053	1.22 [0.99, 1.51]	0.056
Q4	1.59 [1.31, 1.92]	<0.001	1.48 [1.18, 1.86]	0.001	1.48 [1.18, 1.87]	0.001
Per one-unit	1.35 [1.18, 1.54]	<0.001	1.25 [1.07, 1.47]	0.005	1.27 [1.08, 1.50]	0.004

Model 1 was adjusted for age and gender.

Model 2 was adjusted for age, gender, race, smoking, drinking, physical activity, BMI, TC, TG, CRP, and creatinine.

Model 3 was adjusted for age, gender, race, smoking, drinking, physical activity, BMI, TC, TG, CRP, creatinine, glucose, glycated hemoglobin, and insulin.

**Figure 2:**
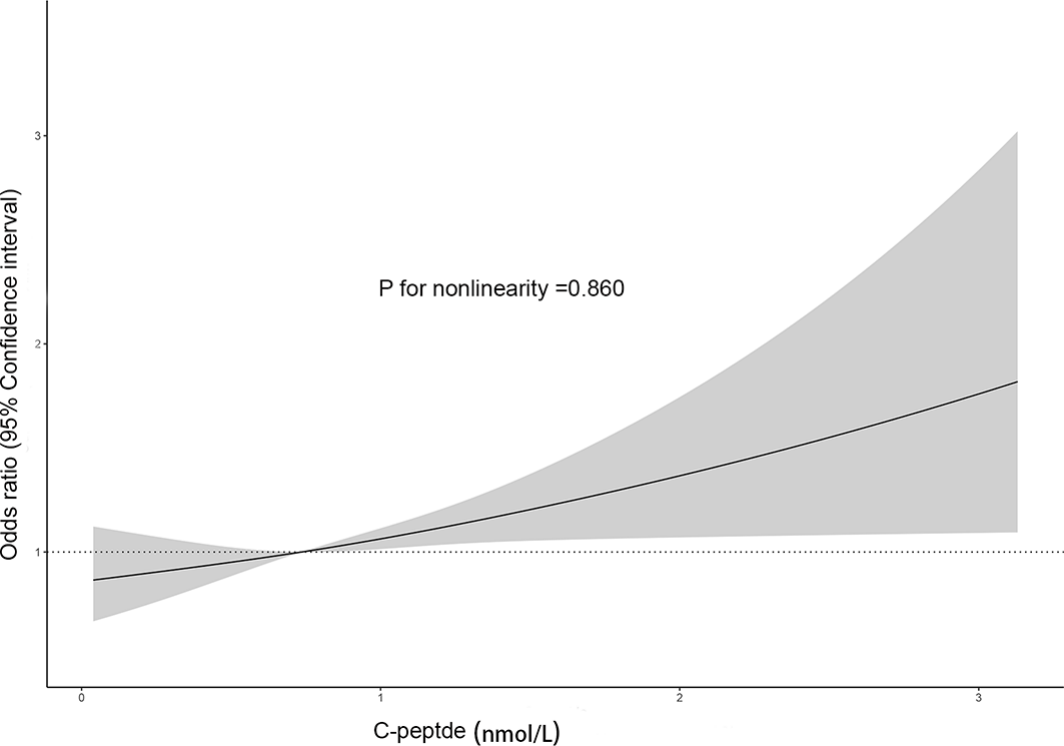
The dose–response relation between C-peptide and SC-MI.

To explore the nonlinear relationship, we performed dose–response analysis based on restricted cubic spline models (**Figure 2**). It suggested that C-peptide was linearly and positively related to SC-MI (*p* for nonlinearity = 0.860).

### Subgroup Analysis

Subgroup analysis was performed to explore the potential factors modifying the association between C-peptide and SC-MI. The association of C-peptide with SC-MI was consistent across age and gender (**Table 4**). Besides, we found that a higher level of C-peptide increased the risk of SC-MI across the disease severity (**Table 5**). However, it was only significant in the borderline abnormality group (*p* = 0.031).

**Table 4:** Subgroup analysis between C-peptide and SC-MI.

	OR (95% CI)	*p*	*p* for interaction
Gender			0.153
Male	1.26 [1.00, 1.62]	0.062	
Female	1.21 [0.95, 1.53]	0.121	
Age			0.262
≤65	1.43 [1.12, 1.84]	0.005	
>65	1.21 [0.97, 1.57]	0.113	

**Table 5:** The association between C-peptide and severity of SC-MI.

Disease severity	Cases	OR (95%CI)	P
Borderline abnormality; 10<CIIS≤15	671	1.24 [1.02, 1.51]	0.031
Possible injury; 15<CIIS≤20	391	1.12 [0.90, 1.37]	0.293
Probable injury; CIIS>20	273	1.15 [0.89, 1.45]	0.259

## Discussion

Our study confirmed that circulating C-peptide level was independently associated with electrocardiographic subclinical myocardial injury after adjusting for cardiovascular risk factors and glucose metabolism-related biomarkers.

Previous studies have shown the association between C-peptide and cardiovascular disease (19). Michelle et al. examined the effect of C-peptide on atherosclerosis and found that high C-peptide levels were related with increased lipid deposits and smooth muscle cell proliferation in the vessel wall, contributing to atherosclerosis (12). Antonio Cabrera de Leon et al. found that elevated C-peptide was associated with the incidence of myocardial infarction and coronary artery disease in the general population (20). What is more, Min et al. showed an association between serum C-peptide levels and all-cause and cause-specific mortality among adults without diabetes (21). Furthermore, we have observed a strong association of C-peptide levels with CIIS and SC-MI in our study, which could contribute to early diagnosis and intervention.

The underlying mechanisms for this association were complicated. The C-peptide has been related to increasing triglyceride levels while decreasing HDL-C levels, which were negatively correlated to cardiovascular death (16). However, evidence also indicated that C-peptide has an anti-inflammatory role (22) and anti-oxidative role (23). So, it needs more prospective clinical trials to determine whether the increased C-peptide in the SC-MI was causal or compensatory.

Certain limitations need to be taken into consideration in the interpretation of our study. Our study was designed by a cross-sectional scheme, only revealing the correlation between C-peptide and SC-MI. Besides, only baseline C-peptide was included in our study, and it may be more meaningful to examine the change of C-peptide.

## Conclusion

We observed an association between high C-peptide levels and SC-MI in the general population.

## Data Availability Statement

The datasets presented in this study can be found in online repositories. The names of the repository/repositories and accession number(s) can be found below: NHANES III.

## Ethics Statement

The studies involving human participants were reviewed and approved by Affiliated Hospital of Nantong University. The patients/participants provided their written informed consent to participate in this study.

## Author Contributions

XMB and MQ designed this study, ZC wrote the manuscript. JH performed the experiments. All authors contributed to the article and approved the submitted version.

## Funding

This study was supported by the Municipal Natural Science Foundation of Nantong (Nos. MS22019004, YYZ-16012 and JCZ20207) and the social science fund of Jiangsu Province (No. 19GLB026).

## Conflict of Interest

The authors declare that the research was conducted in the absence of any commercial or financial relationships that could be construed as a potential conflict of interest.

## Publisher’s Note

All claims expressed in this article are solely those of the authors and do not necessarily represent those of their affiliated organizations, or those of the publisher, the editors and the reviewers. Any product that may be evaluated in this article, or claim that may be made by its manufacturer, is not guaranteed or endorsed by the publisher.

## Abbreviations

SC-MI, subclinical myocardial injury; CIIS, cardiac injury score; NHANES III, National Health and Nutrition Examination Survey III; WBC, white blood cell; RBC, red blood cell; TG, triglyceride; TC, total cholesterol; LDL-C, low-density lipoprotein cholesterol; HDL-C, high-density lipoprotein cholesterol; CRP, C-reactive protein; ALT, alanine aminotransferase; AST, aspartate transaminase glucose; BMI, body mass index.

## References

[1] O’Neal WT Shah AJ Efird JT Rautaharju PM Soliman EZ. Subclinical Myocardial Injury Identified by Cardiac Infarction/Injury Score and the Risk of Mortality in Men and Women Free of Cardiovascular Disease. Am J Cardiol (2014) 114(7):1018–23. 10.1016/j.amjcard.2014.06.032 PMC4336466 25129878

[2] Rubin J Matsushita K Ballantyne CM Hoogeveen R Coresh J Selvin E. Chronic Hyperglycemia and Subclinical Myocardial Injury. J Am Coll Cardiol (2012) 59(5):484–9. 10.1016/j.jacc.2011.10.875 PMC3267958 22281251

[3] Rautaharju PM Warren JW Jain U Wolf HK Nielsen CL. Cardiac Infarction Injury Score: An Electrocardiographic Coding Scheme for Ischemic Heart Disease. Circulation (1981) 64(2):249–56. 10.1161/01.cir.64.2.249 7249294

[4] Richardson K Engel G Yamazaki T Chun S Froelicher VF. Electrocardiographic Damage Scores and Cardiovascular Mortality. Am Heart J (2005) 149(3):458–63. 10.1016/j.ahj.2004.06.025 15864234

[5] German C Ahmad MI Li Y Soliman EZ. Relations Between Physical Activity, Subclinical Myocardial Injury, and Cardiovascular Mortality in the General Population. Am J Cardiol (2020) 125(2):205–9. 10.1016/j.amjcard.2019.08.031 31847957

[6] Vasim I Ahmad MI Mongraw-Chaffin M Soliman EZ. Association of Obesity Phenotypes With Electrocardiographic Subclinical Myocardial Injury in the General Population. Clin Cardiol (2019) 42(3):373–8. 10.1002/clc.23155 PMC6712312 30652323

[7] Waits GS O’Neal WT Sandesara PB Li Y Shah AJ Soliman EZ. Association Between Low Diastolic Blood Pressure and Subclinical Myocardial Injury. Clin Res Cardiol (2018) 107(4):312–8. 10.1007/s00392-017-1184-0 29164391

[8] Ahmad MI Chevli PA Li Y Soliman EZ. Vitamin D Deficiency and Electrocardiographic Subclinical Myocardial Injury: Results From National Health and Nutrition Examination Survey-III. Clin Cardiol (2018) 41(11):1468–73. 10.1002/clc.23078 PMC6489797 30239028

[9] Liu Y Wu M Xu J Sha D Xu B Kang L. Association Between Triglyceride and Glycose (TyG) Index and Subclinical Myocardial Injury. Nutr Metab Cardiovasc Dis (2020) 30(11):2072–6. 10.1016/j.numecd.2020.06.019 32863082

[10] Shaw JA Shetty P Burns KD Fergusson D Knoll GA. C-Peptide as a Therapy for Kidney Disease: A Systematic Review and Meta-Analysis. PloS One (2015) 10(5):e0127439. 10.1371/journal.pone.0127439 25993479 PMC4439165

[11] Yosten GL Maric-Bilkan C Luppi P Wahren J. Physiological Effects and Therapeutic Potential of Proinsulin C-Peptide. Am J Physiol Endocrinol Metab (2014) 307(11):E955–68. 10.1152/ajpendo.00130.2014 PMC4254984 25249503

[12] Alves MT Ortiz MMO Dos Reis G Dusse LMS Carvalho MDG Fernandes AP. The Dual Effect of C-Peptide on Cellular Activation and Atherosclerosis: Protective or Not? Diabetes Metab Res Rev (2019) 35(1):e3071. 10.1002/dmrr.3071 30160822

[13] Patel N Taveira TH Choudhary G Whitlatch H Wu WC. Fasting Serum C-Peptide Levels Predict Cardiovascular and Overall Death in Nondiabetic Adults. J Am Heart Assoc (2012) 1(6):e003152. 10.1161/JAHA.112.003152 23316320 PMC3540682

[14] Abdullah A Hasan H Raigangar V Bani-Issa W. C-Peptide Versus Insulin: Relationships With Risk Biomarkers of Cardiovascular Disease in Metabolic Syndrome in Young Arab Females. Int J Endocrinol (2012) 2012:420792. 10.1155/2012/420792 22899917 PMC3415197

[15] Harnishsingh B Rama B. Is C-Peptide a Predictor of Severity of Coronary Artery Disease in Metabolic Syndrome? An Observational Study. Indian Heart J (2018) 70 Suppl 3:S105–9. 10.1016/j.ihj.2018.07.005 PMC6309290 30595240

[16] Li Y Zhao D Li Y Meng L Enwer G. Serum C-Peptide as a Key Contributor to Lipid-Related Residual Cardiovascular Risk in the Elderly. Arch Gerontol Geriatr (2017) 73:263–8. 10.1016/j.archger.2017.05.018 28869884

[17] Bhatt MP Lim YC Hwang J Na S Kim YM Ha KS. C-Peptide Prevents Hyperglycemia-Induced Endothelial Apoptosis Through Inhibition of Reactive Oxygen Species-Mediated Transglutaminase 2 Activation. Diabetes (2013) 62(1):243–53. 10.2337/db12-0293 PMC3526059 22923476

[18] Liao S Zhang J Shi S Gong D Lu X Cheang I. Association of Aldehyde Exposure With Cardiovascular Disease. Ecotoxicol Environ Saf (2020) 206:111385. 10.1016/j.ecoenv.2020.111385 33010595

[19] Vasic D Walcher D. C-Peptide: A New Mediator of Atherosclerosis in Diabetes. Mediators Inflamm (2012) 2012:858692. 10.1155/2012/858692 22547909 PMC3321614

[20] Cabrera de Leon A Oliva Garcia JG Marcelino Rodriguez I Almeida Gonzalez D Aleman Sanchez JJ Brito Diaz B. C-Peptide as a Risk Factor of Coronary Artery Disease in the General Population. Diabetes Vasc Dis Res (2015) 12(3):199–207. 10.1177/1479164114564900 25696117

[21] Li Y Li Y Meng L Zheng L. Association Between Serum C-Peptide as a Risk Factor for Cardiovascular Disease and High-Density Lipoprotein Cholesterol Levels in Nondiabetic Individuals. PloS One (2015) 10(1):e112281. 10.1371/journal.pone.0112281 25559358 PMC4283961

[22] Kao RLC Xu X Xenocostas A Parry N Mele T Martin CM. C-Peptide Attenuates Acute Lung Inflammation in a Murine Model of Hemorrhagic Shock and Resuscitation by Reducing Gut Injury. J Trauma Acute Care Surg (2017) 83(2):256–62. 10.1097/TA.0000000000001539 28452895

[23] Ragy MM Ahmed SM. Protective Effects of Either C-Peptide or L-Arginine on Pancreatic Beta-Cell Function, Proliferation, and Oxidative Stress in Streptozotocin-Induced Diabetic Rats. J Cell Physiol (2019) 234(7):11500–10. 10.1002/jcp.27808 30515793

